# Lactate to albumin ratio and 30-day mortality in ICU patients with influenza: a single-center retrospective cohort

**DOI:** 10.1186/s12985-026-03230-1

**Published:** 2026-06-28

**Authors:** Weichang Luan, Hongyi Chen, Chunhua Liu, Jingjing Liu, Yanan Qin

**Affiliations:** 1 Emergency Department, Liaocheng People’s Hospital, Liaocheng, China; 2 Department of Infectious Diseases, Liaocheng People’s Hospital, Liaocheng, China

**Keywords:** Lactate-to-albumin ratio, Influenza, ICU, Prognostic value, Mortality

## Abstract

**Background:**

The influenza virus exerts a substantial impact on morbidity, mortality, and overall disease burden among the population, particularly among intensive care unit (ICU) patients. Although multiple biomarkers have been studied for mortality risk stratification in this population, the prognostic relationship of the lactate-to-albumin ratio (LAR) remains incompletely defined in critically ill patients with influenza. This study aims to investigate whether baseline ICU admission LAR levels are independently associated with mortality in critically ill patients with influenza cases and assess its incremental predictive value beyond established scoring systems.

**Methods:**

Utilizing data from the Medical Information Mart for Intensive Care IV database (MIMIC-IV, version 3.1), this retrospective cohort study enrolled adult patients diagnosed with influenza who required ICU admission. LAR was calculated based on lactate and albumin levels measured within 24 h of ICU admission. The primary outcome was 30-day mortality, with secondary outcomes including in-hospital, 60-day, 90-day, and 1-year mortality. The association between LAR and mortality risk was assessed using univariate and multivariate Cox proportional hazards regression models, with robustness evaluated through subgroup analyses and sensitivity analyses. Survival differences across LAR levels were compared via Kaplan-Meier curves and log-rank tests. Discriminative performance was assessed using receiver operating characteristic (ROC) curves and the C-index.

**Results:**

A total of 142 critically ill patients with influenza were included, with an overall 30-day mortality of 22.53%. Univariate Cox regression analysis demonstrated that a higher LAR was associated with an increased risk of 30-day mortality in ICU patients with influenza (HR 2.43, 95% CI 1.80–3.29; *P* < 0.001). After adjusting for confounding factors, multivariable Cox proportional hazards analysis confirmed this association, showing a similarly elevated risk (HR 2.93, 95% CI 1.95–4.39; *P* < 0.001). Kaplan-Meier analysis indicated a substantially lower 30-day survival for the high-LAR group (Log-rank *P* < 0.0001). The AUC of LAR for predicting mortality was 0.783, comparable to APS III (AUC 0.785, 95% CI 0.696–0.874; *P* = 0.140), SAPS II (AUC 0.717, 95% CI 0.617–0.817; *P* = 0.157), OASIS (AUC 0.643, 95% CI 0.531–0.756; *P* = 0.168), and SOFA (AUC 0.687, 95% CI 0.580–0.794; *P* = 0.160). Notably, the AUC for albumin alone was 0.791, compared with 0.783 for LAR.

**Conclusion:**

This study found that early LAR upon ICU admission is independently associated with the 30-day mortality rate in critically ill patients with influenza. Its predictive ability seems to be comparable to established clinical scoring systems. However, LAR did not outperform albumin alone, and its added prognostic value beyond albumin remains uncertain, and further validation in prospective studies is still needed.

**Supplementary Information:**

The online version contains supplementary material available at https://doi.org/10.1186/s12985-026-03230-1.

## Introduction

 Influenza remains a significant global public health burden, with seasonal epidemics resulting in substantial morbidity and mortality [1, 2]. According to the World Health Organization, approximately 1 billion influenza infections occur globally each year, with 3 to 5 million resulting in severe seasonal influenza illnesses [3, 4]. According to data from the U.S. Centers for Disease Control and Prevention, influenza has caused between 9.3 million and 41 million illnesses and 120,000 to 710,000 hospitalizations annually [5]. Some patients, particularly children, the elderly, pregnant women, and individuals with underlying medical conditions, may progress to severe influenza. This condition is characterized by complications including viral pneumonia, acute respiratory distress syndrome (ARDS), and multi-organ dysfunction, often requiring intensive care unit (ICU) admission and potentially leading to death from influenza-related complications [6–10]. Despite advances in supportive care, influenza patients admitted to the ICU continue to face a high risk of mortality. For example, a study from southern Sweden reported a one-year mortality rate as high as 43% among adults admitted to the ICU with influenza A or B [11]. Therefore, early identification and risk stratification of severe influenza patients are essential for optimizing ICU resource allocation and improving clinical outcomes.

In recent years, the lactate-to-albumin ratio (LAR) has emerged as a novel indicator reflecting the severity of infection, with its prognostic utility reported in various critical care settings. Lactate is a product of anaerobic metabolism, which can accurately reflect the degree of cellular hypoxia and insufficient tissue perfusion, and it is a marker of the severity of shock [12]. In contrast, albumin, as a negative acute-phase reaction protein, decreases during severe inflammation and critical illness due to capillary leakage, reduced synthesis, increased decomposition, and blood dilution caused by fluid therapy [13]. The ratio formed by the combination of the two can enhance the prognostic assessment efficacy by integrating metabolic stress and systemic inflammatory conditions. A number of studies have shown that the LAR level at admission serves as an independent predictor of mortality in individuals suffering from sepsis and its related organ dysfunction diseases, with some studies demonstrating superior prognostic performance compared with using lactate or albumin alone [14–18]. Notably, Liu et al. prospectively validated LAR’s prognostic accuracy in a single-center sepsis trial, implementing prespecified measurement protocols and demonstrating robust performance [19]. Beyond sepsis, LAR serves as a robust predictor of mortality across multiple critical conditions, including trauma and acute pancreatitis [20, 21]. However, the association of this simple and effective prognostic tool in the specific high-risk population of critically ill patients with influenza has not been fully explored. While a recent study [8] by Gao reported preliminary associations between LAR and mortality in influenza-associated ARDS, its analysis was limited to univariate assessments without comprehensive adjustment for confounders or evaluation of incremental prognostic utility.

Therefore, this study used data from the large public database Medical Information Mart for Intensive Care IV (MIMIC-IV, version 3.1) to comprehensively evaluate the relationship of LAR among critically ill adults with influenza through a retrospective cohort study. The existing literature on prognostic markers of influenza-associated critical illness mostly focuses on single parameters or complex scoring systems, and there is limited attention to composite ratios such as LAR that are easy to obtain. We proposed that higher levels of LAR upon ICU admission correlate independently with greater mortality risk among critically ill patients with influenza. Its predictive ability seems to be comparable to that of established severity scoring systems.

## Methods

### Data source

Patient data were retrieved from the publicly accessible Medical Information Mart for Intensive Care database (MIMIC-IV, version 3.1; https://doi.org/10.13026/kpb9-mt58). This comprehensive single-center repository encompasses records of patients admitted to the emergency department or ICU at Beth Israel Deaconess Medical Center in Boston. It contains retrospective data from over 65,000 ICU stays and more than 200,000 emergency department encounters spanning the period from 2008 to 2019 [22]. Access authorization for the database was granted by PhysioNet to one author (Yanan Qin) following the completion of mandatory online training and assessment (CITI certification number 70873436). Because the database is anonymized, informed consent was not required according to the data use agreement and institutional policy. This retrospective cohort study was conducted in accordance with the Strengthening the Reporting of Observational Studies in Epidemiology (STROBE) statement [23].

### Study population

**Fig. 1:**
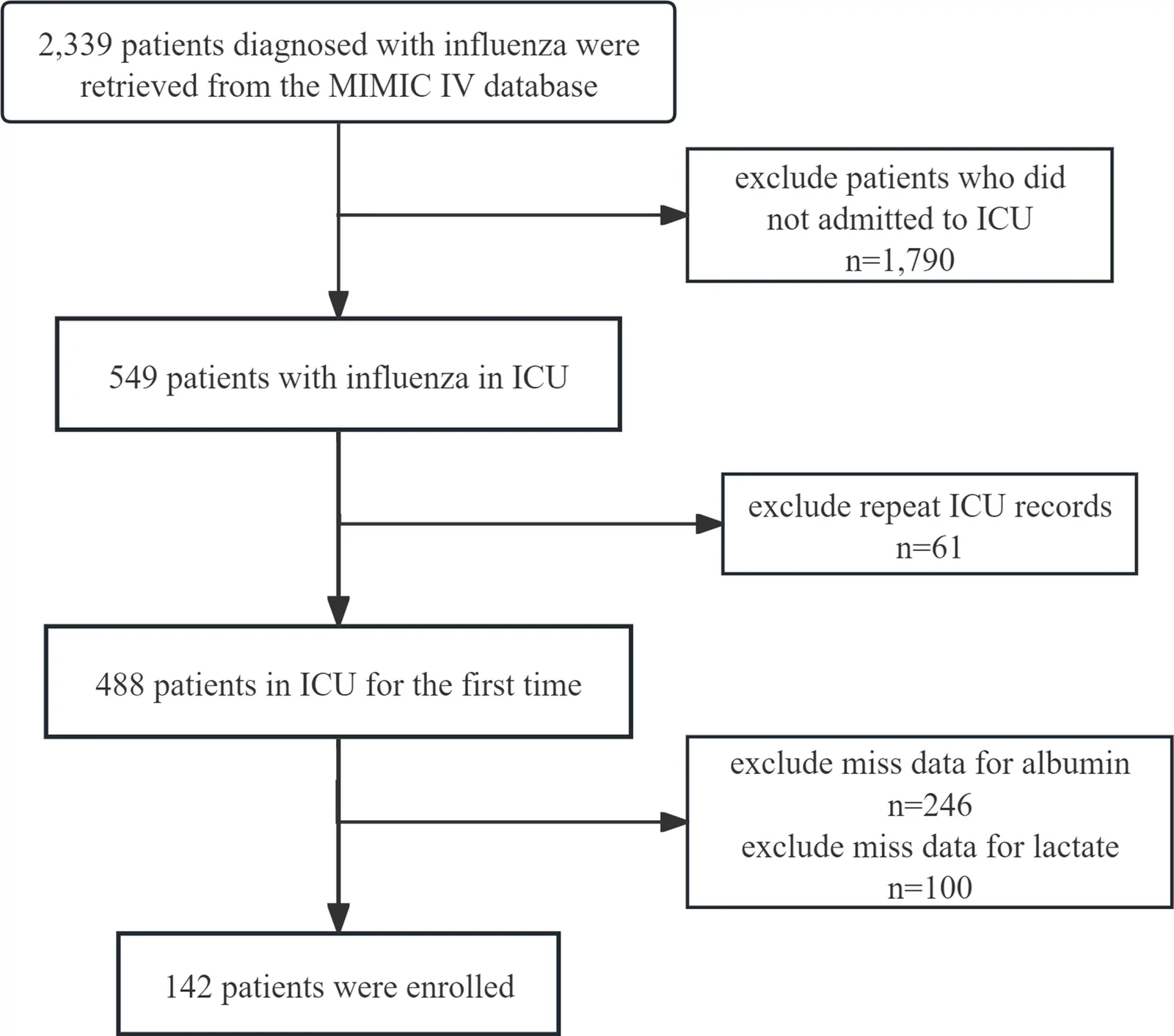
Flowchart of study population inclusion and exclusion. MIMIC-IV, Medical Information Mart for Intensive Care; ICU, intensive care unit

Within the MIMIC-IV database, this study first screened all individuals aged 18 years or older with influenza during the period from 2008 to 2022. Influenza diagnoses were based on the International Classification of Diseases, Ninth and Tenth Revisions (codes 487×, 488×, J09, J09×, J10×, J100×, and J11×) [24]. Patients were excluded based on the following criteria: (1) patients without ICU admission records; (2) patients with repeated ICU admissions during the same hospitalization—only the first ICU admission record was retained; (3) patients who lacked serum lactate or albumin measurements within 24 h after ICU admission. Notably, because lactate and albumin were essential predictors, patients with missing values for either measurement within 24 h of ICU admission were excluded, which may introduce selection bias. Finally, 142 patients were included, and the specific inclusion and exclusion process is shown in Fig. 1.

### Variable collection

Utilizing structured SQL queries, we retrieved essential patient records from the MIMIC-IV database and archived them in PostgreSQL. Captured parameters encompassed core demographic variables including age, gender, race, marital status, height, and weight; vital signs including heart rate, blood pressure (systolic, diastolic, and mean pulse pressure), respiratory rate, body temperature, and peripheral blood oxygen saturation; comorbidities (sepsis, congestive heart failure, myocardial infarction, chronic pulmonary disease, hypertension, diabetes, renal disease, malignant cancer, severe liver disease, metastatic solid tumors, and Charlson Comorbidity Index); treatments (vasoactive agent use, ventilation, antibiotics, and antiviral drug use); severity clinical scores (Acute Physiology Score III (APS III), Simplified Acute Physiology Score II (SAPS II), Oxford Acute Severity of Illness Score (OASIS), Sequential Organ Failure Assessment (SOFA), Glasgow Coma Scale (GCS), and Systemic Inflammatory Response Syndrome (SIRS)); laboratory parameters, including lactate and albumin for LAR calculation (values extracted within 24 h of ICU admission), along with white blood cell (WBC) and differential, platelet, hematocrit, hemoglobin, red cell distribution width, prothrombin time (PT), international normalized ratio (INR), alanine aminotransferase (ALT), aspartate aminotransferase (AST), alkaline phosphatase, total bilirubin (TBIL), albumin, blood urea nitrogen, creatinine, electrolytes (calcium, potassium, sodium, chloride), creatine kinase, lactate dehydrogenase, aniongap, bicarbonate, and glucose; patient clinical outcomes (length of hospital/ICU stay and mortality at 30, 60, 90, and 365 days) were also extracted. Notably, for all vital signs and laboratory measurements, we extracted the mean value of all recordings obtained within the first 24 h after ICU admission to represent the patient’s initial physiological status. Baseline vital signs, laboratory values, and clinical scoring data were defined as those recorded within 24 h of ICU admission. Covariates with > 20% missing data (Table S1) were excluded from analysis. Variables with ≤ 20% missing data (Table S2-3) underwent multiple imputation. Multiple imputation using Fully Conditional Specification (FCS) implemented by the MICE algorithm as described in Van Buuren and Groothuis-Oudshoorn (2011) https://doi.org/10.18637/jss.v045.i03IF: 8.1 Q1 B2 [25], with random-forest imputation (five imputations). The full list of variables included in the imputation model is provided in Table S3. Final regression estimates were pooled according to Rubin’s rules.

## Outcome

This study’s main endpoint was 30-day all-cause mortality after ICU admission. Secondary endpoints comprised in-hospital mortality post-ICU admission, along with overall mortality at 60-day, 90-day, and 1-year time points.

### Statistical analysis

Continuous variables were for normality using the Shapiro-Wilk test. Data conforming to normal distribution were expressed as mean ± standard deviation (SD) and analyzed with the Student’s t-test. Non-normally distributed data were presented as medians with interquartile range (IQR) and compared using the Mann-Whitney U test. Categorical variables were presented as frequencies and percentages compared using the Chi-square test or Fisher’s exact test, as appropriate.

Multivariable logistic regression was employed to assess the association between LAR and in-hospital mortality. Odds ratios (OR) with corresponding 95% confidence intervals (CI) were reported. Univariate and multivariate Cox proportional hazards models were employed to evaluate associations between LAR and 30-day, 60-day, 90-day, and 1-year mortality. Hazard ratios (HR) with corresponding 95% confidence intervals (CI) were reported. The proportional hazards assumption was verified using Schoenfeld residuals and was adequately met. Two models were constructed: Model 1 represented unadjusted analysis, while Model 2 adjusted for SOFA score, OASIS score, aniongap, blood urea nitrogen, and hemoglobin. Variance inflation factors (VIFs) were ≤ 2, indicating no significant multicollinearity (Table S4). Covariate selection for Model 2 was predicated on clinical relevance, prior literature, and statistical criteria. Kaplan-Meier curves illustrated 30-day survival differences between LAR strata, with log-rank tests evaluating intergroup significance. LAR was evaluated both as a continuous measure (per unit increase) and as a categorical variable. For categorical analysis, including the description of baseline characteristics, multivariate Cox proportional hazards regression models, and Kaplan-Meier (K-M) survival curves, LAR was dichotomized at the cohort median: Q1 (LAR < 0.485) and Q2 (LAR ≥ 0.485), with Q1 serving as the reference group.

To explore the potential of LAR as a prognostic biomarker and identify a cutoff value optimized for predictive performance regarding the primary outcome(s) within our cohort, ROC curve analysis was performed. The optimal cutoff point was determined using the Youden index, maximizing the sum of sensitivity and specificity. Discriminatory performance of established prognostic scores (APS III, SAPS II, OASIS, and SOFA) was evaluated both individually and in combination with LAR. Area under the curve (AUC) with 95% CI was calculated via DeLong’s method, and statistical comparisons of paired models utilized DeLong’s nonparametric test. Model calibration was evaluated using the Brier score, where lower values indicate better predictive accuracy. Discriminative performance was assessed using the concordance index (C-index), while model improvement was quantified via integrated discrimination improvement (IDI), net reclassification improvement (NRI), and decision curve analysis (DCA).

To assess the robustness of our findings, we performed a series of sensitivity analyses. First, to evaluate the potential influence of extreme values of the LAR, we excluded observations with LAR values below the 1st percentile or above the 99th percentile and re-estimated the primary regression model. Second, to address the impact of missing data, we conducted a complete-case analysis, restricting the sample to participants with complete data on all covariates included in the primary model. Third, to examine the stability of the covariate selection, we applied best subset regression (BSR) using the Bayesian information criterion (BIC). The best-fitting model was identified as the one with the lowest BIC value, and the primary exposure effect was re-estimated using the covariates selected by this procedure. Subgroup analyses via stratified Cox regression assessed the consistency of LAR’s prognostic effect across clinically relevant subgroups, retaining core covariates from the primary analysis.

Statistical analyses were executed with R4.2.2 (The R Foundation for Statistical Computing; http://www.R-project.org*)* and Free Statistics 2.4. The statistical significance threshold was defined as a two-tailed *p*-value below 0.05.

## Results

1. **Baseline characteristics of the study population**

Among the 142 included patients, the 30-day mortality was 22.53% (*n* = 32). Baseline characteristics stratified by LAR are presented in Tables 1 and 2. Patients in the high LAR group showed increased heart rate (*P* < 0.001), decreased SBP (*P* = 0.001), and a higher prevalence of congestive heart failure (*P* = 0.012); conversely, the prevalence of hypertension was lower (*P* = 0.018). Significant differences were also observed in laboratory parameters: patients in the high LAR group had higher WBC, PT, INR, AST, TBIL, aniongap, and lactate levels and lower albumin levels (all *P* < 0.05). In addition, they showed higher disease severity scores, such as APS III, SOFA, and SIRS scores (all *P* < 0.05); and faced higher risks of mortality outcomes (*P* < 0.001).

**Table 1:** Baseline clinical and laboratory characteristics of patients stratified by LAR group

Variables	Total (*N* = 142)	Q1 (*n* = 71, 50%)	Q2 (*n* = 71, 50%)	*P*-value
Sex/male, n (%)	76 (53.5)	39 (54.9)	37 (52.1)	0.736
Age(years)	65.1 ± 16.5	65.1 ± 16.7	65.0 ± 16.4	0.967
Marital/married, n (%)	66 (46.5)	29 (40.8)	37 (52.1)	0.339
Race/white, n (%)	79 (55.6)	41 (57.7)	38 (53.5)	0.612
LOS(icu), days	3.8 (2.1, 10.3)	4.7 (2.0, 11.2)	3.3 (2.1, 8.1)	0.184
Heart rate (bpm)	92.0 ± 17.7	85.7 ± 16.3	98.3 ± 16.8	**< 0.001**
SBP (mmHg)	115.6 ± 15.2	119.7 ± 14.1	111.5 ± 15.2	**0.001**
DBP (mmHg)	65.5 ± 11.0	65.5 ± 11.6	65.6 ± 10.4	0.98
MAP (mmHg)	79.4 ± 10.9	79.7 ± 11.5	79.2 ± 10.4	0.777
Resp rate (bpm)	21.9 ± 4.6	21.5 ± 4.3	22.4 ± 4.9	0.279
Temperature (℃)	37.0 ± 0.6	37.1 ± 0.6	37.0 ± 0.6	0.436
SpO_2_ (%)	95.9 ± 2.9	95.5 ± 2.4	96.3 ± 3.3	0.098
Congestive heart failure, n (%)	65 (45.8)	25 (35.2)	40 (56.3)	**0.012**
Chronic pulmonary disease, n (%)	63 (44.4)	35 (49.3)	28 (39.4)	0.237
Hypertension, n (%)	43 (30.3)	28 (39.4)	15 (21.1)	**0.018**
Diabetes, n (%)	43 (30.3)	18 (25.4)	25 (35.2)	0.201
Renal disease, n (%)	36 (25.4)	15 (21.1)	21 (29.6)	0.247
Malignant cancer, n (%)	21 (14.8)	10 (14.1)	11 (15.5)	0.813
Charlson comorbidity index	5.8 ± 3.1	5.5 ± 2.8	6.2 ± 3.2	0.153
Sepsis, n (%)	116 (81.7)	59 (83.1)	57 (80.3)	0.664
White blood cell (K/uL)	10.3 (6.2, 14.3)	8.8 (5.8, 12.3)	11.7 (7.0, 16.1)	**0.025**
Lymphocytes (K/uL)	1.0 (0.5, 27.4)	1.0 (0.6, 31.3)	0.9 (0.5, 5.5)	0.512
Neutrophils (K/uL)	8.0 (4.5, 14.6)	7.1 (4.6, 9.8)	8.9 (4.5, 18.3)	0.152
Hemoglobin (g/dL)	10.6 ± 2.1	10.6 ± 2.1	10.6 ± 2.2	0.869
Platelet (K/uL)	196.0 ± 114.3	196.5 ± 102.5	195.6 ± 125.9	0.965
Prothrombin time (s)	14.4 (12.3, 17.3)	12.9 (11.8, 15.0)	16.0 (13.2, 22.4)	**< 0.001**
International normalized ratio	1.3 (1.1, 1.6)	1.2 (1.1, 1.4)	1.5 (1.2, 2.0)	**< 0.001**
Alanine aminotransferase (U/L)	27.0 (18.0, 52.4)	24.0 (18.0, 40.8)	30.5 (18.0, 62.0)	0.102
Aspartate aminotransferase (U/L)	40.0 (27.6, 82.5)	35.0 (25.0, 65.0)	48.0 (29.5, 124.2)	**0.047**
Total bilirubin (mg/dL)	0.4 (0.3, 0.8)	0.4 (0.3, 0.6)	0.6 (0.3, 0.9)	**0.005**
Albumin (g/dL)	3.1 ± 0.7	3.2 ± 0.6	2.9 ± 0.7	**0.001**
Blood urea nitrogen (mg/dL)	23.5 (15.5, 42.4)	21.5 (15.0, 40.5)	29.5 (17.0, 44.2)	0.183
Creatinine (mg/dL)	1.2 (0.8, 2.2)	1.1 (0.8, 1.6)	1.4 (0.9, 2.3)	0.136
Aniongap (mmol/L)	15.8 ± 4.4	14.8 ± 4.2	16.9 ± 4.5	**0.004**
Bicarbonate (mmol/L)	22.8 ± 5.2	23.5 ± 4.6	22.1 ± 5.7	0.118
Lactate (mmol/L)	1.4 (1.0, 2.1)	1.0 (0.8, 1.3)	2.1 (1.6, 3.0)	**< 0.001**
Glucose (mg/dL)	160.2 ± 74.2	149.1 ± 52.8	171.3 ± 89.7	0.073
Scoring system				
APS III	64.1 ± 27.0	59.5 ± 26.1	68.6 ± 27.2	**0.042**
SAPS II	42.5 ± 15.6	40.0 ± 14.4	45.0 ± 16.5	0.057
OASIS	37.7 ± 9.8	36.9 ± 9.8	38.5 ± 9.8	0.312
SOFA	3.0 (2.0, 5.0)	2.0 (2.0, 4.0)	3.0 (2.0, 6.0)	**0.025**
GCS	11.2 ± 4.1	10.8 ± 4.2	11.6 ± 3.9	0.205
SIRS	2.9 ± 0.9	2.7 ± 0.9	3.0 ± 0.8	**0.016**
Ventilation, n (%)	94 (66.2)	47 (66.2)	47 (66.2)	1.000
Vasoactive, n (%)	66 (46.5)	30 (42.3)	36 (50.7)	0.313

LAR, lactate-to-albumin ratio; LOS, length of stay; SBP, systolic blood pressure; DBP, diastolic blood pressure; MAP, mean arterial pressure; SpO_2_, saturation of peripheral oxygen; APS III, Acute Physiology Score III, SAPS II, Simplified Acute Physiology Score II; OASIS, Oxford Acute Severity of Illness Score; SOFA, Sequential Organ Failure Assessment; GCS, Glasgow Coma scale; SIRS, systemic inflammatory response syndrome. Bold values in Tables 1 indicate statistical significance (*p* < 0.05)

**Table 2:** Outcomes of patients stratified by LAR group

Variables	Total (*N* = 142)	Q1 (*n* = 71, 50%)	Q2 (*n* = 71, 50%)	*P*-value
In-icu death, n (%)	19 (13.4)	2 (2.8)	17 (23.9)	**< 0.001**
In-hospital death, n (%)	29 (20.4)	5 (7.0)	24 (33.8)	**< 0.001**
30 day mortality, n (%)	32 (22.5)	5 (7.0)	27 (38.0)	**< 0.001**
60 day mortality, n (%)	36 (25.4)	8 (11.3)	28 (39.4)	**< 0.001**
90 day mortality, n (%)	40 (28.2)	10 (14.1)	30 (42.3)	**< 0.001**
365 day mortality, n (%)	57 (40.1)	19 (26.8)	38 (53.5)	**0.001**

LAR, lactate-to-albumin ratio. Bold values in Tables 2 indicate statistical significance (*p* < 0.05)

2. **Association of LAR with 30-day mortality**

Analysis of univariate Cox regression models (Table S5) revealed that LAR, lactate, albumin, total bilirubin, lymphocyte count, hemoglobin, glucose, heart rate, resp rate, disease severity scores (including APS III, SAPS II, OASIS, and SOFA), and Charlson comorbidity index were associated with 30-day mortality (all *P* < 0.05). Analysis of multivariate Cox regression models (Table 3) revealed that elevated LAR was significantly associated with increased 30-day mortality when unadjusted (HR 2.43, 95% CI 1.80–3.29; *P* < 0.001). This association remained significant after adjusting for SOFA score, OASIS score, aniongap, blood urea nitrogen, and hemoglobin (HR 2.93, 95% CI 1.95–4.39; *P* < 0.001). After full adjustment, patients in Q2 had a higher risk of 30-day mortality compared with Q1 (HR 6.59, 95% CI 2.47–17.56; *P* < 0.001). Schoenfeld residuals confirmed proportional hazards assumptions (Tables S6–S7).

**Fig. 2:**
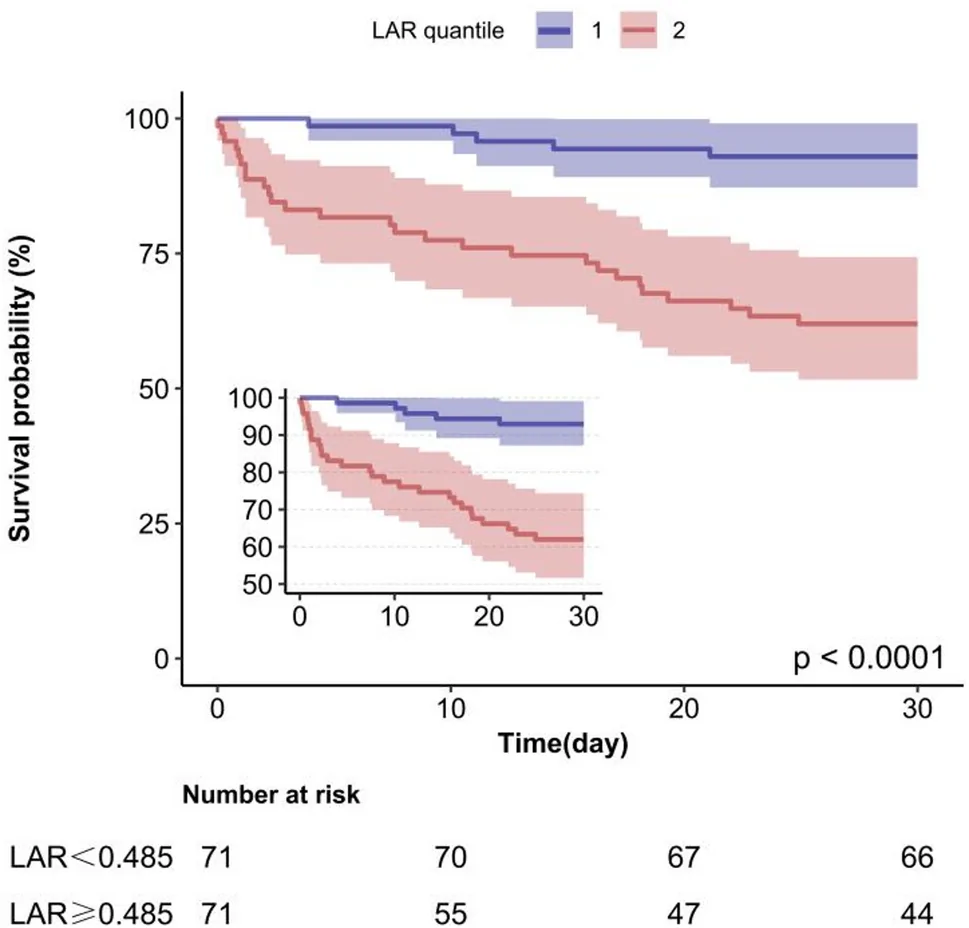
Kaplan-Meier survival curves for ICU patients with influenza stratified by LAR levels. LAR, lactate-to-albumin ratio; ICU, intensive care unit

Kaplan-Meier analysis showed that patients in the high LAR group had significantly worse 30-day mortality than those in the low LAR group (log-rank *P* < 0.001) (Fig. 2).

**Table 3:** Multivariable cox regression analysis of LAR associated with 30-day mortality in ICU patients with influenza

Variables	Event rate	Model 1		Model 2	
		HR (95%CI)	*P*-value	HR (95%CI)	*P*-value
LAR	32/142	2.43 (1.80 ~ 3.29)	< 0.001	2.93 (1.95 ~ 4.39)	< 0.001
LAR(categorical)					
Q1	5/71	1(Ref)		1(Ref)	
Q2	27/71	6.60 (2.54 ~ 17.15)	< 0.001	6.59 (2.47 ~ 17.56)	< 0.001

LAR, lactate-to-albumin ratio; ICU, intensive care unit; HR, hazard ratio; CI, confidence interval

Model 1: Unadjusted

Model 2: Adjusted for SOFA score, OASIS score, aniongap, blood urea nitrogen, hemoglobin

3. **Predictive performance and incremental value of LAR**

The predictive performance of LAR and established scoring systems is summarized in Table 4. The optimal cut-off value of LAR for predicting the 30-day mortality outcome of critically ill patients with influenza was 0.456. Based on this threshold, patients were stratified into low-LAR and high-LAR groups. The mortality rate was 3.1% in the low-LAR group versus 39% in the high-LAR group. LAR exhibited moderate discriminatory ability for mortality prediction (AUC 0.783, 95% CI 0.702–0.865). Calibration, as assessed by the Brier score, was favorable for LAR (0.153) and best for albumin (0.139). Decision curve analysis showed that LAR provided relatively higher net benefit across a wide range of threshold probabilities compared with established scoring systems (Figure S1).

When integrated with established scoring systems, LAR significantly enhanced mortality predictive performance, evidenced by increased C-index values and statistically significant IDI and NRI (Table S8). As demonstrated by the decision curve analysis, LAR offered limited incremental benefit to established severity scores when used as a continuous variable (Figure S2) but significantly improved the net benefit of all scores when incorporated as a categorical variable (Figure S3).

4. **Sensitivity and subgroup analyses**

**Fig. 3:**
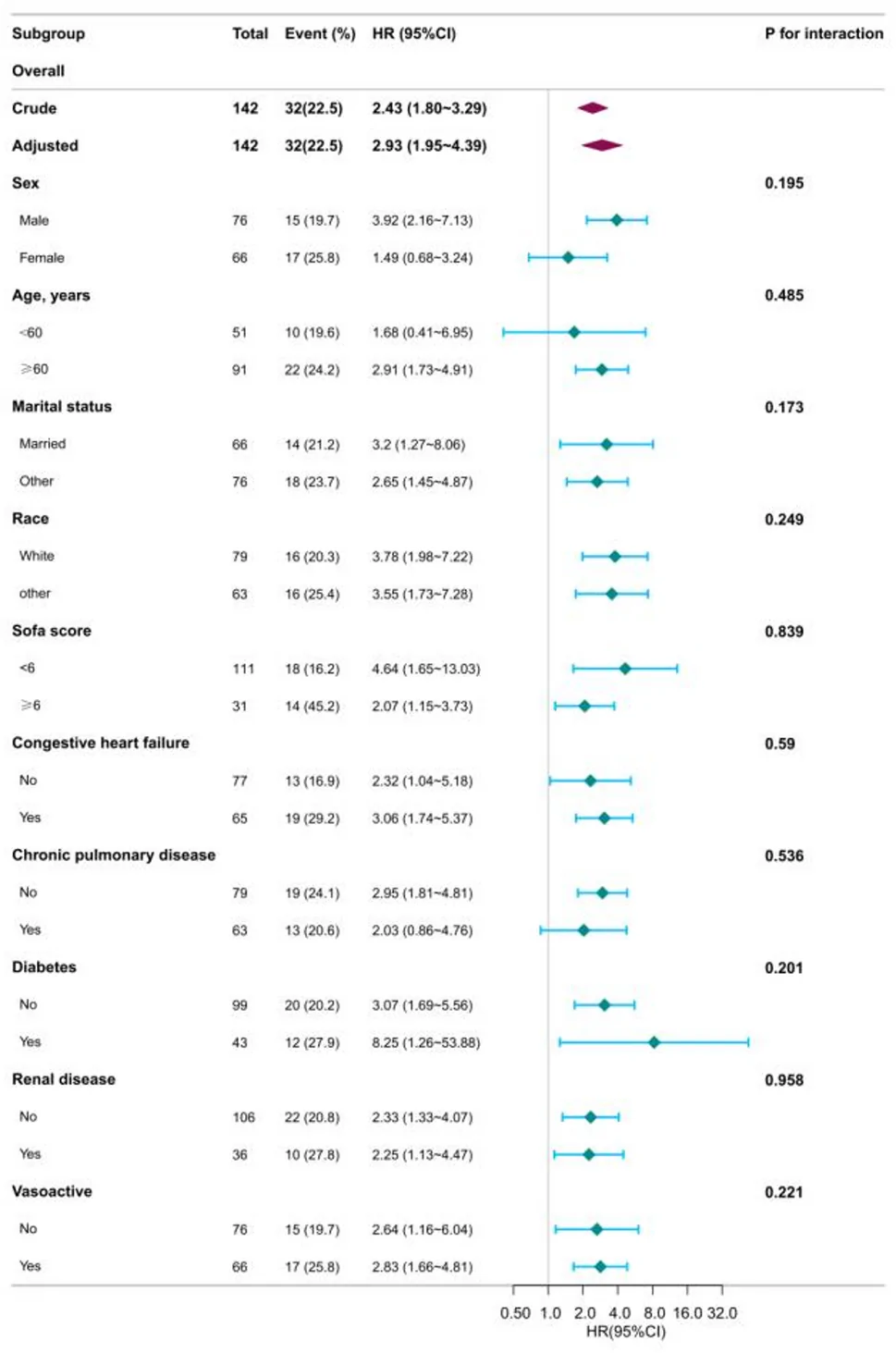
Subgroup analyses of the association between LAR and 30-day mortality in ICU patients with influenza. HR, hazard ratio; CI, confidence interval; SOFA, Sequential Organ Failure Assessment; LAR, lactate-to-albumin ratio; ICU, intensive care unit

The results of the sensitivity analyses were consistent with those of the primary analysis. After excluding observations with extreme LAR values (below the 1st percentile or above the 99th percentile), the estimated effect remained similar in magnitude and statistical significance (Table S9). The complete-case analysis yielded comparable results to the primary analysis, which had applied multiple imputation for missing covariates (Table S10). Finally, after applying BIC-based best subset regression for covariate selection, the effect estimate for the primary exposure did not materially change, supporting the robustness of the model specification (Table S11). Subgroup analysis indicated that the positive correlation between elevated LAR and increased mortality risk was consistent in most subgroups, confirming its predictive robustness (Fig. 3).

**Table 4:** ROC curves comparing predictive performance of LAR versus conventional severity scores and aniongap in critically ill patients with influenza

Variables	AUC(95%CI)	Brier score	*P* for comparison	Optimal cutoff value	Sensitivity	Specificity	NPV	PPV	Youden index
LAR	0.783 (0.702, 0.865)	0.153	Ref.	0.456	0.938	0.573	0.969	0.390	0.510
APS III	0.785 (0.696, 0.874)	0.140	0.978	63.500	0.844	0.664	0.935	0.422	0.507
SAPS II	0.717 (0.617, 0.817)	0.157	0.313	38.500	0.906	0.509	0.949	0.349	0.415
OASIS	0.643 (0.531, 0.756)	0.168	0.055	39.500	0.625	0.636	0.854	0.333	0.261
SOFA	0.687 (0.580, 0.794)	0.160	0.129	5.500	0.438	0.846	0.838	0.452	0.283
GCS	0.603 (0.483, 0.723)	0.169	0.037	12.500	0.625	0.609	0.848	0.317	0.234
SIRS	0.548 (0.444, 0.652)	0.173	< 0.001	2.500	0.750	0.318	0.814	0.242	0.068
Albumin	0.791 (0.711, 0.871)	0.139	0.872	3.250	0.938	0.500	0.965	0.353	0.438
Lactate	0.706 (0.613, 0.800)	0.169	< 0.001	1.450	0.750	0.618	0.895	0.364	0.368
Aniongap	0.581 (0.472, 0.690)	0.173	< 0.001	12.750	0.875	0.291	0.889	0.264	0.166

ROC, receiver operating characteristic; LAR, lactate-to-albumin ratio; AUC, area under the curve; CI, confidence interval; NPV, negative predictive value; PPV, positive predictive value; APS III, Acute Physiology Score III; SAPS II, Simplified Acute Physiology Score II; OASIS, Oxford Acute Severity of Illness Score; SOFA, Sequential Organ Failure Assessment; GCS, Glasgow Coma scale; SIRS, systemic inflammatory response syndrome

5. **Secondary outcomes**

Elevated LAR demonstrated significant associations with increased in-hospital mortality (Table 5). Moreover, it was consistently associated with heightened risks of 60-day, 90-day, and 1-year mortality (Table 6).

**Table 5:** Multivariable logistic regression analysis of LAR associated with in-hospital mortality in ICU patients with influenza

Variable	Event rate	Model 1		Model 2	
		OR (95%CI)	*P*-value	OR (95%CI)	*P*-value
LAR	29/142	4.12 (1.82 ~ 9.3)	0.001	5.51 (1.86 ~ 16.39)	0.002
LAR(categorical)					
Q1	5/71	1(Ref)		1(Ref)	
Q2	24/71	6.74 (2.4 ~ 18.95)	< 0.001	8.24 (2.46 ~ 27.61)	0.001

LAR, lactate-to-albumin ratio; ICU, intensive care unit; OR, odds ratios; CI, confidence interval

Model 1: Unadjusted

Model 2: Adjusted for SOFA score, OASIS score, aniongap, blood urea nitrogen, hemoglobin

**Table 6:** Multivariable cox regression analysis of LAR associated with 60-day, 90-day, and 1-year mortality in ICU patients

Variable	Event rate	Model 1		Model 2	
		HR (95%CI)	*P*-value	HR (95%CI)	*P*-value
60 day mortality					
LAR	36/142	2.33 (1.73 ~ 3.14)	< 0.001	2.75 (1.85 ~ 4.07)	< 0.001
LAR(categorical)					
Q1	8/71	1(Ref)		1(Ref)	
Q2	28/71	4.38 (1.99 ~ 9.62)	< 0.001	4.32 (1.93 ~ 9.65)	< 0.001
90 day mortality					
LAR	40/142	2.26 (1.68 ~ 3.03)	< 0.001	2.63 (1.78 ~ 3.89)	< 0.001
LAR(categorical)					
Q1	10/71	1(Ref)		1(Ref)	
Q2	30/71	3.81 (1.86 ~ 7.8)	< 0.001	3.8 (1.83 ~ 7.91)	< 0.001
1 year mortality					
LAR	57/142	2.17 (1.64 ~ 2.86)	< 0.001	2.43 (1.71 ~ 3.44)	< 0.001
LAR(categorical)					
Q1	19/71	1(Ref)		1(Ref)	
Q2	38/71	2.66 (1.53 ~ 4.62)	0.001	2.91 (1.65 ~ 5.13)	< 0.001

LAR, lactate-to-albumin ratio; ICU, intensive care unit; HR, hazard ratio; CI, confidence interval

Model 1: Unadjusted

Model 2: Adjusted for SOFA score, OASIS score, aniongap, blood urea nitrogen, hemoglobin

## Discussion

This research systematically assessed the association between the LAR and mortality in critically ill patients with influenza. We demonstrated that LAR measured at ICU admission is independently associated with the 30-day mortality in this population. Regarding predictive performance, LAR exhibited moderate discriminatory ability for 30-day mortality (AUC: 0.783; 95% CI: 0.702–0.865), which was statistically comparable to that of albumin alone (AUC 0.791, 95%CI 0.711–0.871; *P* = 0.872) and to several established severity scores, including APS III (AUC 0.785, 95%CI 0.696–0.874; *P* = 0.978), SAPS II (AUC 0.717, 95%CI 0.617–0.817; *P* = 0.313), OASIS (AUC 0.643, 95%CI 0.531–0.756; *P* = 0.055), and SOFA (AUC 0.687, 95%CI 0.580–0.794; *P* = 0.129). When integrated with conventional severity scores, LAR was associated with observed changes in reclassification metrics such as IDI and NRI. However, due to the small sample size, multiple comparisons, and the absence of internal validation procedures, these reclassification statistics should be interpreted with extreme caution and are considered hypothesis-generating only; they do not provide reliable evidence of incremental prognostic value. Decision curve analysis offered a more nuanced but similarly limited perspective: while LAR added as a continuous variable showed negligible incremental net benefit, its incorporation as a categorical variable based on the optimal cut-off suggested a possible net benefit in some models. Nevertheless, the APS III score alone consistently achieved the highest net benefit across a broad range of threshold probabilities, underscoring the continued relevance of comprehensive scoring systems. Taken together, these exploratory findings suggest that LAR is a readily available biomarker associated with mortality in this cohort, with performance comparable to some complex severity scores and superior to certain traditional indicators. However, given the near-identical performance of albumin alone, the marginal difference observed in our cohort suggests that the incremental prognostic utility of LAR specifically over albumin remains uncertain, we interpret these results with appropriate caution and warrant further investigation in larger, independent datasets.

Regarding prognostic factors for severe influenza, age and comorbidities (such as cardiopulmonary diseases, immunocompromised status, and malignancies) represent significant nonmodifiable host factors [9, 10, 26, 27]. Advanced age itself constitutes a well-established risk factor for influenza severity, while “biological age”—reflecting functional physiological status—may hold greater predictive significance than chronological age. Research indicates that novel aging biomarkers characterizing cellular senescence, immunosenescence, and systemic inflammation (such as epigenetic clocks, telomere length, and specific inflammatory cytokine profiles) have demonstrated potential in predicting influenza severity and vaccination efficacy [28]. However, the clinical application of these biomarkers remains largely confined to research settings and has not yet been integrated into routine clinical practice. Concerning clinical assessment tools, traditional critical illness scoring systems such as SOFA, which integrate multiple physiological parameters, have been validated for effectively evaluating disease severity and mortality risk in influenza patients [8, 29]. Nevertheless, their relatively complex data collection requirements limit rapid deployment during early disease stages. Meanwhile, single laboratory parameters often exhibit limited prognostic value. For instance, a MIMIC database study revealed that aniongap demonstrated weaker predictive capability for severe influenza outcomes (AUC 0.598) compared to SOFA (AUC 0.673) and SAPS II scores (AUC 0.766), likely attributable to its reflection of only partial acid-base equilibrium information [24].

LAR, as a composite indicator integrating both “stress” and “reserve” information, reflects the combined nutritional-inflammatory profile of critically ill cases. This methodology diminishes the impact of patient-specific variables on immune regulatory processes, positioning the LAR as a reliable prognostic indicator for diverse pathological conditions. The core pathology of severe influenza involves a systemic disorder resulting from both direct viral replication-induced cellular damage and host hyperimmune responses (termed “cytokine storm”) [3]. In severe influenza patients, elevated lactate levels stem from the combined effects of direct viral action, immune responses, tissue hypoxia, and hepatic/renal dysfunction. Mechanistically, influenza A virus infection upregulates lactate dehydrogenase A (LDHA) expression in host cells, promoting lactate production [30]. Beyond serving as a metabolic byproduct, lactate actively contributes to viral immune evasion by binding to mitochondrial antiviral signaling protein (MAVS), thereby inhibiting RIG-I-mediated type I interferon responses—a mechanism that appears specific to influenza infection [30]. Concurrently, virus-triggered inflammatory responses further enhance aerobic glycolysis, increasing lactate generation. Additionally, severe influenza patients frequently develop hypoxemia, driving cells toward anaerobic metabolism for energy production. This intensified anaerobic glycolysis further elevates lactate levels. Impaired hepatic and renal function reduces lactate clearance capacity, leading to lactate accumulation. Collectively, these mechanisms cause significantly elevated lactate levels in severe influenza patients, exacerbating disease severity [30]. Thus, hyperlactatemia reflects not only hypoxia but also systemic inflammatory intensity and cellular energy crisis. Simultaneously, hypoalbuminemia reveals depletion of homeostatic reserve: during intense systemic inflammation, albumin synthesis is suppressed in the liver as it is a negative acute-phase protein. Concurrently, inflammation-induced capillary leakage causes substantial albumin loss into interstitial spaces, compounded by hypercatabolic states, collectively resulting in hypoalbuminemia [21]. Importantly, influenza infection has been documented to directly trigger systemic capillary leak syndrome (SCLS), characterized by profound hypoalbuminemia secondary to endothelial injury and capillary hyperpermeability, which further exacerbates the severity of illness [31, 32]. Therefore, low albumin levels comprehensively reflect uncontrolled systemic inflammation and severe endothelial barrier disruption. These dual pathological processes create a vicious cycle in critically ill patients: hypoalbuminemia exacerbates tissue edema, further impairing oxygen delivery and perpetuating lactate elevation. The value of LAR lies in its simultaneous quantification of both the intensity of “acute metabolic stress” and the degree of depletion in “chronic physiological reserve/inflammatory burden.” This dual assessment provides a more comprehensive, mechanistically grounded early warning signal for identifying high-risk patients.

A previous study utilizing a self-constructed database demonstrated that LAR exhibits excellent predictive capability for progression to ARDS in influenza A pneumonia patients (AUC 0.878). For predicting 28-day mortality, it achieved an AUC of 0.881 with 89.7% specificity, albeit relatively lower sensitivity (73.3%) [8]. However, these findings warrant further validation and refinement. First, the limited sample size (*n* = 105) necessitates verification in larger cohorts. More critically, the mortality analysis employed only univariate regression without adjusting for key confounders such as disease severity scores, potentially introducing confounding bias. In contrast, our study implemented rigorous multivariate Cox proportional hazards regression within a public database. After adjusting for critical covariates, including SOFA and OASIS scores, etc., we confirmed LAR as an independent predictor of mortality in ICU influenza patients, enhancing result validity and clarifying its independent prognostic value. Furthermore, LAR demonstrated sustained predictive power across multiple mortality timepoints, indicating prognostic significance beyond short-term outcomes and supporting dynamic risk surveillance and long-term management. Through direct comparison with established scoring systems, our findings further support the potential utility of LAR in this clinical context. Notably, LAR’s AUC was significantly higher than that of lactate alone (e.g., lactate AUC = 0.706, *p* < 0.05), indicating improved diagnostic performance over another widely used single biomarker. Although albumin alone showed a numerically higher AUC (0.791 vs. 0.783 for LAR), the difference was not statistically significant (*p* = 0.872), and albumin levels in critically ill patients are influenced by multiple factors (inflammation, capillary leak, nutrition, and liver function), which may limit its stability as a standalone marker. Crucially, this study also examined whether adding LAR to conventional severity scores (e.g., SOFA or SAPS II) was associated with changes in predictive performance. In our small dataset, ROC analysis showed numerical trends toward improved AUC when LAR was combined with these scores. However, the clinical translation of this finding requires careful contextualization. Our analysis was restricted to patients with complete biomarker data available within 24 h of ICU admission, which excluded approximately 70% of otherwise eligible patients. As excluded patients exhibited significantly lower illness severity and mortality, our findings may be most applicable to critically ill populations meeting strict monitoring criteria typical of tertiary-care ICUs. Given the comparable performance of albumin alone, the lack of internal validation, and the exploratory nature of all incremental performance analyses, these potential applications warrant further validation in larger, prospective cohorts before clinical implementation recommendations can be made.

Furthermore, our findings should be considered within the broader context of viral critical illness, where the pathophysiology is often compounded by secondary insults. Secondary bacterial infections are well-recognized contributors to adverse outcomes in severe viral pneumonia. ICU-based studies in SARS-CoV-2 populations have demonstrated that secondary infections significantly increase morbidity and mortality, highlighting the complex interplay between viral injury, immune dysregulation, and bacterial complications [33]. In this context, a composite inflammatory–metabolic marker such as LAR may partially reflect the cumulative burden of these viral and bacterial insults, integrating the metabolic stress of critical illness with the nutritional and inflammatory consequences of infection. The potential utility of simple, aggregated biomarkers is further supported by research in broader septic populations. Readily available hematological indices such as the neutrophil-to-lymphocyte ratio and platelet-related parameters have shown independent prognostic value and potential complementarity to established severity scores [34]. These findings reinforce the broader concept that simple laboratory-derived composite markers may contribute meaningfully to risk stratification in critically ill infectious diseases.

However, limitations exist within this investigation. First, selection bias is a concern. Approximately 70% of otherwise eligible patients were excluded because lactate or albumin measurements were not available within 24-hour of ICU admission. Excluded patients were significantly less severely ill, as indicated by shorter ICU stays, lower severity scores, and lower rates of sepsis (Table S12). However, the primary reason for missingness was our strict 24-hour time window rather than selective testing of sicker patients: albumin—a routine test—had a higher missing rate (50.4%) than lactate (20.5%), and many patients had these tests performed before ICU admission. Nonetheless, the net effect is that our findings overrepresent more severe cases and cannot be generalized to all ICU patients with influenza, especially those with lower illness severity or tests performed exclusively pre-admission. Second, due to the retrospective observational design, residual confounding from unmeasured variables—such as antiviral therapy, viral load, or influenza subtype—cannot be ruled out, despite the use of multivariable regression and subgroup analyses. Third, the use of a small sample and a single-center database (MIMIC-IV) limits external validity. The absence of an external validation cohort means our findings, while internally rigorous, require independent confirmation before any claims of generalizability can be made. Fourth, the reliance on ICD-10 codes for influenza case identification, rather than universal PCR or antigen testing, represents a methodological limitation. While ICD coding offers practical advantages for large-scale retrospective ICU studies involving critically ill patients—where routine laboratory confirmation is often incomplete—this approach carries the potential for misclassification bias. Fifth, the sample size and events-per-variable (EPV) ratio were limited. We did not perform bootstrapping, cross-validation, or penalized regression; therefore, all performance estimates (including AUC, IDI, NRI, and decision curve analysis) are likely optimistic. Sixth, although LAR emerged as an independent predictor of mortality, its discriminatory ability was not superior to albumin alone (AUC: 0.783 vs. 0.791, *p* = 0.872). This suggests that the predictive contribution of LAR in this cohort may be largely driven by albumin, with lactate offering limited incremental value. Seventh, LAR may be confounded by bacterial superinfection or antibiotic use, limiting its specificity for influenza-related severity. Future studies should incorporate microbiological data to isolate viral-specific effects. Finally, our analysis relied on a single admission-day LAR measurement; dynamic changes in lactate or albumin over time—which may offer additional prognostic insight—were not assessed. Prospective studies with serial biomarker sampling are needed to evaluate temporal trajectories and their clinical utility.

Despite these limitations, our findings hold significant clinical implications and potential generalizability. The calculation of LAR requires only two routine blood test parameters—cost-effective and rapidly available—making it suitable for integration into ICU influenza admission screening protocols. It can serve as either a risk-stratification tool for early high-risk identification or an effective complement to complex scoring systems. Based on this research, future investigations should focus on: (1) Implementing multi-center prospective cohorts to assess LAR’s prognostic utility and define optimized risk-stratification thresholds; (2) Incorporating LAR into influenza clinical decision pathways to prospectively assess its impact on treatment optimization and patient outcomes; (3) Combining LAR with emerging biomarkers (such as cytokines and genomic markers) and artificial intelligence algorithms (such as deep learning architectures) to develop more precise, personalized prognostic prediction models.

## Conclusion

This study suggests that the LAR measured early at ICU admission may be associated with short- and long-term mortality in critically ill patients with influenza. Our findings indicate that LAR demonstrates discriminatory ability comparable to that of established severity scores. However, it is noteworthy that albumin alone had an AUC of 0.791, compared with 0.783 for LAR. This finding indicates that the added prognostic value of LAR over albumin alone remains uncertain. Therefore, LAR should be viewed only as an exploratory biomarker at this stage, not as a clinical tool for decision-making. Prospective, multicenter studies with larger and more diverse cohorts are warranted to externally validate these findings and to rigorously assess whether LAR provides clinically meaningful added value beyond established markers like albumin before any clinical application can be considered.

## Supplementary Information

Supplementary material 1.

## Data Availability

No datasets were generated or analysed during the current study.

## Abbreviations

AUC: Area under the curve
ARDS: Acute respiratory distress syndrome
APS III: Acute physiology score III
ALT: Alanine aminotransferase
AST: Aspartate aminotransferase
BSR: Best subset regression
BIC: Bayesian information criterion
CI: Confidence intervals
DCA: Decision curve analysis
DBP: Diastolic blood pressure
EPV: Events-per-variable
FCS: Fully conditional specification
GCS: Glasgow coma scale
HR: Hazard ratios
ICU: Intensive care unit
IQR: Interquartile range
INR: International normalized ratio
IDI: Integrated discrimination improvement
LAR: Lactate-to-albumin ratio
LOS: Length of stay
MIMIC-IV: Medical information mart for intensive care IV
MAP: Mean arterial pressure
NRI: Net reclassification improvement
NPV: Negative predictive value
OASIS: Oxford acute severity of illness score
OR: Odds ratios
PT: Prothrombin time
PPV: Positive predictive value
ROC: Receiver operating characteristic
STROBE: Strengthening the reporting of observational studies in epidemiology
SBP: Systolic blood pressure
SAPS II: Simplified acute physiology score II
SOFA: Sequential organ failure assessment
SIRS: Systemic inflammatory response syndrome
SD: Standard deviation
SpO2: Saturation of peripheral oxygen
TBIL: Total bilirubin
WBC: White blood cell
