# Supplementary material for: Lactate to albumin ratio and 30-day mortality in ICU patients with influenza: a single-center retrospective cohort
Source: Virol J. 2026 Jun 28;23:211. doi: 10.1186/s12985-026-03230-1 (PMC13573348; doi:10.1186/s12985-026-03230-1)
Supplement: Supplementary file 1 [file 12985_2026_3230_MOESM1_ESM.docx]

**Supplementary Tables**

**Table S1** Variables with > 20% missing data and missing percentages.

| **Variables** | **Missing data percentage** |
| --- | --- |
| LDH | 36.6% |
| Height | 40.8% |
| CK-MB | 50.7% |
| CK | 51.4% |
| Fibrinogen | 73.9% |

LDH, lactate dehydrogenase; CK-MB, creatine kinase-MB; CK, creatine kinase

**Table S2** Variables with < 20% missing data and missing percentages.

| **Variables** | **Missing data percentage** |
| --- | --- |
| Insurance | 0.7% |
| Temperature | 0.7% |
| Glucose | 1.4% |
| Weight | 1.40% |
| Alanine aminotransferase | 3.5% |
| Alkaline phosphatase | 3.5% |
| Aspartate aminotransferase | 3.5% |
| Total bilirubin | 4.9% |
| Prothrombin time | 5.6% |
| International normalized ratio | 5.6% |
| Lymphocytes | 12.7% |
| Neutrophils | 12.7% |
| Marital | 12.7% |
| SOFA | 18.3% |

SOFA, Sequential Organ Failure Assessment

**Table S3** Variables included in the multiple imputation model.

| Sex | Diabetes | Total bilirubin | 90 day mortality |
| --- | --- | --- | --- |
| Age | Malignant cancer | Albumin | 365 day mortality |
| LOS(icu) | Charlson comorbidity index | Blood urea nitrogen | Scoring system |
| Heart rate | Sepsis | Creatinine | APS III |
| SBP | White blood cell | Aniongap | SAPS II |
| DBP | Lymphocytes | Bicarbonate | OASIS |
| MAP | Neutrophils | Lactate | SOFA |
| Resp rate | Hemoglobin | Glucose | GCS |
| Temperature | Platelet | In-icu death | SIRS |
| SpO2 | Prothrombin time | In-hospital death | Ventilation |
| Congestive heart failure | International normalized ratio | 28 day mortality | Vasoactive |
| Chronic pulmonary disease | Alanine aminotransferase | 30 day mortality | Oseltamivir phosphate |
| Hypertension | Aspartate aminotransferase | 60 day mortality | Antibiotic |

LOS, length of stay; SBP, systolic blood pressure; DBP, diastolic blood pressure; MAP, mean arterial pressure; SpO2, saturation of peripheral oxygen; APS III, Acute Physiology Score III, SAPS II, Simplified Acute Physiology Score II; OASIS, Oxford Acute Severity of Illness Score; SOFA, Sequential Organ Failure Assessment; GCS, Glasgow Coma scale; SIRS, systemic inflammatory response syndrome

**Table S4** VIFs of covariables

| **Variables** | **VIFs** |
| --- | --- |
| SOFA | 1.455 |
| OASIS | 1.548 |
| Aniongap | 1.806 |
| Blood urea nitrogen | 1.954 |
| Hemoglobin | 1.189 |

VIFs, Variance inflation factors; SOFA, Sequential Organ Failure Assessment; OASIS, Oxford Acute Severity of Illness Score

**Table S5** Univariate cox regression analysis of LAR associated with 30-day mortality in critically ill patients with influenza

| **Variables** | **HR(95%CI)** | ***P*-value** | **Variables** | **HR(95%CI)** | ***P*-value** |
| --- | --- | --- | --- | --- | --- |
| Sex | 1.31 (0.65, 2.62) | 0.445 | LAR | 2.43 (1.80, 3.29) | < 0.001 |
| Age | 1.01 (0.98, 1.03) | 0.599 | White blood cell | 1.00 (0.98, 1.02) | 0.821 |
| Race | 1.24 (0.62, 2.48) | 0.546 | Lymphocytes | 0.99 (0.99, 1.00) | 0.048 |
| Marital | 1.29 (0.59, 2.83) | 0.764 | Neutrophils | 1.00 (0.99, 1.01) | 0.488 |
| LOS(icu) | 0.98 (0.94, 1.03) | 0.455 | Hemoglobin | 0.77 (0.64, 0.92) | 0.003 |
| Heart rate | 1.03 (1.01, 1.05) | 0.003 | Platelet | 1.00 (0.99, 1.00) | 0.478 |
| SBP | 0.98 (0.95, 1.01) | 0.131 | Prothrombin time | 1.02 (1.00, 1.04) | 0.134 |
| DBP | 0.99 (0.96, 1.03) | 0.635 | International normalized ratio | 1.13 (0.94, 1.36) | 0.264 |
| MAP | 0.99 (0.96, 1.03) | 0.583 | Alanine aminotransferase | 1.00 (0.99, 1.00) | 0.352 |
| Resp rate | 1.12 (1.03, 1.20) | 0.005 | Aspartate aminotransferase | 1.00 (1.00, 1.00) | 0.058 |
| Temperature | 0.56 (0.32, 1.00) | 0.055 | Total bilirubin | 1.23 (1.07, 1.43) | 0.030 |
| SpO2 | 0.91 (0.81, 1.03) | 0.177 | Albumin | 0.16 (0.09, 0.3) | < 0.001 |
| Congestive heart failure | 1.87 (0.92, 3.78) | 0.079 | Blood urea nitrogen | 1.01 (1.00, 1.02) | 0.065 |
| Chronic pulmonary disease | 0.79 (0.39, 1.61) | 0.517 | Creatinine | 1.01 (0.93, 1.08) | 0.886 |
| Hypertension | 0.86 (0.40, 1.86) | 0.702 | Aniongap | 1.06 (1.00, 1.13) | 0.096 |
| Diabetes | 1.47 (0.72, 3.00) | 0.304 | Bicarbonate | 0.99 (0.93, 1.06) | 0.828 |
| Renal disease | 1.44 (0.68, 3.05) | 0.347 | Lactate | 1.19 (1.05, 1.34) | 0.026 |
| Malignant cancer | 1.05 (0.40, 2.73) | 0.920 | Glucose | 1.01 (1.00, 1.01) | 0.007 |
| Charlson comorbidity index | 1.16 (1.05, 1.28) | 0.007 | Ventilation | 1.33 (0.61, 2.87) | 0.463 |
| Sepsis | 1.22 (0.47, 3.18) | 0.673 | Vasoactive | 1.37 (0.68, 2.74) | 0.377 |
| APS III | 1.03 (1.02, 1.04) | < 0.001 | SOFA | 1.22 (1.11, 1.34) | < 0.001 |
| SAPS II | 1.04 (1.02, 1.06) | < 0.001 | GCS | 0.92 (0.86, 1.00) | 0.051 |
| OASIS | 1.05 (1.01, 1.09) | 0.012 | SIRS | 2.59 (0.32, 21.08) | 0.373 |

LAR, lactate-to-albumin ratio; HR,hazard ratio; CI,confidence interval; LOS, length of stay; SBP, systolic blood pressure; DBP, diastolic blood pressure; MAP, mean arterial pressure; SpO2, saturation of peripheral oxygen; APS III, Acute Physiology Score III; SAPS II, Simplified Acute Physiology Score II; OASIS, Oxford Acute Severity of Illness Score; SOFA, Sequential Organ Failure Assessment; GCS, Glasgow Coma scale; SIRS, systemic inflammatory response syndrome

**Table S6** Schoenfeld residual tests for proportional hazards (LAR-specific), outcome: 30-day mortality

| **Model** | **Chi-square** | **df** | ***P*-value** |
| --- | --- | --- | --- |
| Model 1 | 0.624 | 1 | 0.429 |
| Model 2 | 0.881 | 1 | 0.348 |

LAR, lactate-to-albumin ratio; df, Degrees of freedom

**Table S7** Schoenfeld residual tests for proportional hazards (global), outcome: 30-day mortality

| **Model** | **Chi-square** | **df** | ***P*-value** |
| --- | --- | --- | --- |
| Model 1 | 0.624 | 1 | 0.429 |
| Model 2 | 11.224 | 6 | 0.082 |

df, Degrees of freedom

**Table S8** Incremental prognostic value of LAR for predicting 30-day mortality

| **Variables** | **C-index(95%CI)** | ***P* for comparison** | **IDI(95%CI)** | ***P*-value** | **NRI(95%CI)** | | ***P*-value** |
| --- | --- | --- | --- | --- | --- | --- | --- |
| LAR(continuous) |  |  |  |  |  | |  |
| APS III | 0.785 (0.696, 0.874) |  | Ref. |  | Ref. | |  |
| APS III+LAR | 0.826 (0.747, 0.904) | 0.053 | 0.091 (0.019, 0.198) | 0.016 | 0.261 (-0.038, 0.468) | | 0.056 |
| SAPS II | 0.717 (0.617, 0.817) |  | Ref. |  | Ref. | |  |
| SAPS II+LAR | 0.810 (0.728, 0.892) | 0.009 | 0.105 (0.018, 0.239) | 0.012 | 0.270 (-0.005, 0.467) | | 0.060 |
| OASIS | 0.643 (0.531, 0.756) |  | Ref. |  | Ref. | |  |
| OASIS+LAR | 0.769 (0.675, 0.862) | 0.006 | 0.136 (0.041, 0.267) | 0.004 | 0.270 (0.073, 0.479) | | 0.020 |
| SOFA | 0.687 (0.580, 0.794) |  | Ref. |  | Ref. | |  |
| SOFA+LAR | 0.778 (0.692, 0.864) | 0.033 | 0.091 (0.030, 0.184) | <0.001 | | 0.202 (0.085, 0.459) | 0.008 |
| LAR(categorical) |  |  |  |  | |  |  |
| APS III | 0.785 (0.696, 0.874) |  | Ref. |  | | Ref. |  |
| APS III+LAR | 0.884 (0.824, 0.944) | 0.004 | 0.135 (0.047, 0.229) | 0.012 | | 0.510 (0.339, 0.616) | 0.004 |
| SAPS II | 0.717 (0.617, 0.817) |  | Ref. |  | | Ref. |  |
| SAPS II+LAR | 0.844 (0.773, 0.915) | 0.001 | 0.156 (0.066, 0.251) | 0.008 | | 0.510 (0.316, 0.616) | 0.008 |
| OASIS | 0.643(0.531, 0.756) |  | Ref. |  | | Ref. |  |
| OASIS+LAR | 0.815 (0.739, 0.891) | <0.001 | 0.170 (0.075, 0.273) | 0.008 | | 0.510 (0.385, 0.618) | 0.004 |
| SOFA | 0.687 (0.580, 0.794) |  | Ref. |  | | Ref. |  |
| SOFA+LAR | 0.845 (0.747, 0.903) | <0.001 | 0.161 (0.063, 0.262) | 0.012 | | 0.510 (0.314, 0.616) | 0.004 |

LAR, lactate-to-albumin ratio; CI, confidence interval; IDI, integrated discrimination improvement; NRI, net reclassification improvement; APS III, Acute Physiology Score III; SAPS II, Simplified Acute Physiology Score II; OASIS, Oxford Acute Severity of Illness Score; SOFA, Sequential Organ Failure Assessment

**Table S9** Multivariable cox regression analysis of LAR associated with 30-day mortality in ICU patients with influenza after excluding extreme values (2% trimmed)

| **Variables** | **Event rate** | **Model 1** | | **Model 2** | |
| --- | --- | --- | --- | --- | --- |
|  |  | HR (95%CI) | *P*-value | HR (95%CI) | *P*-value |
| LAR | 30/138 | 2.22 (1.47~3.36) | <0.001 | 2.50 (1.41~4.44) | 0.002 |
| LAR(categorical) |  |  |  |  |  |
| Q1 | 5/69 | 1(Ref) |  | 1(Ref) |  |
| Q2 | 25/69 | 6.00 (2.30~15.69) | <0.001 | 5.95 (2.24~15.77) | <0.001 |

LAR, lactate-to-albumin ratio; ICU, intensive care unit; HR, hazard ratio; CI, confidence interval

Model 1: Unadjusted

Model 2: Adjusted for SOFA score, OASIS score, aniongap, blood urea nitrogen, hemoglobin

**Table S10** Multivariable cox regression analysis of LAR associated with 30-day mortality in ICU patients with influenza based on complete cases

| **Variables** | **Event rate** | **Model 1** | | **Model 2** | |
| --- | --- | --- | --- | --- | --- |
|  |  | HR (95%CI) | *P*-value | HR (95%CI) | *P*-value |
| LAR | 27/116 | 2.97 (2.05~4.29) | <0.001 | 3.54 (2.18~5.74) | <0.001 |
| LAR(categorical) |  |  |  |  |  |
| Q1 | 5/58 | 1(Ref) |  | 1(Ref) |  |
| Q2 | 22/58 | 5.33 (2.02~14.09) | 0.001 | 4.73 (1.75~12.78) | 0.002 |

LAR, lactate-to-albumin ratio; ICU, intensive care unit; HR, hazard ratio; CI, confidence interval

Model 1: Unadjusted

Model 2: Adjusted for SOFA score, OASIS score, aniongap, blood urea nitrogen, hemoglobin

**Table S11** Multivariable cox regression analysis of LAR associated with 30-day mortality in ICU patients with influenza (BIC-BSR-adjusted Model 2)

| **Variables** | **Event rate** | **Model 1** | | **Model 2** | |
| --- | --- | --- | --- | --- | --- |
|  |  | HR (95%CI) | *P*-value | HR (95%CI) | *P*-value |
| LAR | 32/142 | 2.43 (1.80~3.29) | <0.001 | 2.43 (1.72~3.44) | <0.001 |
| LAR(categorical) |  |  |  |  |  |
| Q1 | 5/71 | 1(Ref) |  | 1(Ref) |  |
| Q2 | 27/71 | 6.60 (2.54~17.15) | <0.001 | 4.52 (1.68~12.20) | 0.003 |

LAR, lactate-to-albumin ratio; ICU, intensive care unit; BIC, bayesian information criterion; BSR, best subset regression; HR, hazard ratio; CI, confidence interval

Model 1: Unadjusted

Model 2: Adjusted for heart rate, charlson comorbidity index, APS III score

**TableS 12** Baseline characteristics of patients stratified by LAR data

| **Variables** | **Total (N = 488)** | **Without LAR data (n = 346)** | **With LAR data (n = 142)** | ***P*-value** |
| --- | --- | --- | --- | --- |
| Sex/male, n (%) | 245 (50.2) | 169 (48.8) | 76 (53.5) | 0.348 |
| Age(years) | 65.7±18.1 | 65.9±18.8 | 65.1±16.5 | 0.642 |
| Marital/married, n (%) | 197 (43.7) | 137 (41.9) | 60 (48.4) | 0.407 |
| Race/white, n (%) | 253 (51.8) | 174 (50.3) | 79 (55.6) | 0.283 |
| LOS(icu), days | 2.5 (1.2, 5.7) | 1.9 (1.0, 4.2) | 3.8 (2.1, 10.3) | < 0.001 |
| Heart rate (bpm) | 90.0±16.9 | 89.2±16.5 | 92.0±17.7 | 0.098 |
| SBP (mmHg) | 118.3±15.2 | 119.4±15.1 | 115.6±15.2 | 0.011 |
| DBP (mmHg) | 66.2±10.7 | 66.4±10.6 | 65.5±11.0 | 0.413 |
| MAP (mmHg） | 79.8±10.6 | 80.0±10.5 | 79.4±10.9 | 0.625 |
| Resp rate (bpm) | 21.6±4.2 | 21.4±4.1 | 21.9±4.6 | 0.213 |
| Temperature (℃) | 37.0±0.5 | 37.0±0.5 | 37.0±0.6 | 0.365 |
| SpO_2_ (%) | 96.0±2.9 | 96.0±2.9 | 95.9±2.9 | 0.608 |
| Congestive heart failure, n (%) | 203 (41.6) | 138 (39.9) | 65 (45.8) | 0.230 |
| Chronic pulmonary disease, n (%) | 210 (43.0) | 147 (42.5) | 63 (44.4) | 0.703 |
| Hypertension, n (%) | 161 (33.0) | 118 (34.1) | 43 (30.3) | 0.415 |
| Diabetes, n (%) | 156 (32.0) | 113 (32.7) | 43 (30.3) | 0.609 |
| Renal disease, n (%) | 125 (25.6) | 89 (25.7) | 36 (25.4) | 0.932 |
| Malignant cancer, n (%) | 80 (16.4) | 59 (17.1) | 21 (14.8) | 0.540 |
| Charlson comorbidity index | 5.9±3.1 | 6.0±3.2 | 5.8±3.1 | 0.656 |
| Sepsis, n (%) | 310 (63.5) | 194 (56.1) | 116 (81.7) | < 0.001 |
| White blood cell (K/uL) | 8.5 (5.9, 12.6) | 8.2 (5.7, 11.9) | 10.3 (6.2, 14.3) | 0.008 |
| lymphocytes (K/uL) | 1.5 (0.6, 46.2) | 2.0 (0.7, 53.8) | 1.0 (0.5, 29.8) | 0.006 |
| Neutrophils (K/uL) | 7.5 (4.5, 12.0) | 7.4 (4.5, 11.9) | 7.9 (4.5, 12.5) | 0.629 |
| Hemoglobin (g/dL) | 10.8±2.2 | 10.9±2.2 | 10.6±2.1 | 0.179 |
| Platelet (K/uL) | 187.3±94.4 | 183.7±84.8 | 196.0±114.3 | 0.190 |
| Prothrombin time (s) | 17.1±10.2 | 16.7±9.0 | 17.9±12.3 | 0.290 |
| international normalized ratio | 1.6±1.0 | 1.5±0.9 | 1.7±1.3 | 0.244 |
| Alanine aminotransferase (U/L) | 25.2 (17.0, 47.0) | 24.5 (16.0, 44.0) | 27.0 (18.0, 52.0) | 0.177 |
| Aspartate aminotransferase (U/L) | 39.0 (26.5, 67.5) | 37.8 (25.0, 60.0) | 41.0 (28.0, 84.0) | 0.101 |
| Total bilirubin (mg/dL) | 0.5 (0.3, 0.8) | 0.5 (0.3, 0.8) | 0.4 (0.3, 0.8) | 0.189 |
| Albumin (g/dL) | 3.2±0.7 | 3.3±0.7 | 3.1±0.7 | 0.009 |
| Blood urea nitrogen (mg/dL) | 20.5 (13.5, 37.5) | 19.5 (12.0, 33.2) | 23.5 (15.5, 42.4) | < 0.001 |
| Creatinine (mg/dL) | 1.1 (0.8, 1.8) | 1.0 (0.8, 1.6) | 1.2 (0.8, 2.2) | 0.018 |
| Aniongap (mmol/L) | 15.5±3.9 | 15.3±3.7 | 15.8±4.4 | 0.188 |
| Bicarbonate (mmol/L) | 23.0±5.1 | 23.1±5.1 | 22.8±5.2 | 0.618 |
| Lactate (mmol/L) | 1.3 (1.0, 2.0) | 1.3 (1.0, 1.9) | 1.4 (1.0, 2.1) | 0.429 |
| Glucose (mg/dL) | 156.6±82.3 | 155.1±85.6 | 160.2±74.2 | 0.539 |
| Scoring system |  |  |  |  |
| APS III | 52.7±24.9 | 48.0±22.4 | 64.1±27.0 | < 0.001 |
| SAPS II | 37.1±14.7 | 34.9±13.7 | 42.5±15.6 | < 0.001 |
| OASIS | 33.6±9.4 | 31.9±8.7 | 37.7±9.8 | < 0.001 |
| SOFA | 3.6±2.2 | 3.5±1.9 | 3.9±2.6 | 0.159 |
| GCS | 12.4±3.6 | 13.0±3.3 | 11.2±4.1 | < 0.001 |
| SIRS | 2.6±0.9 | 2.5±0.9 | 2.9±0.9 | < 0.001 |
| Ventilation, n (%) | 210 (43.0) | 116 (33.5) | 94 (66.2) | < 0.001 |
| Vasoactive, n (%) | 155 (31.8) | 89 (25.7) | 66 (46.5) | < 0.001 |

LAR, lactate-to-albumin ratio; LOS, length of stay; SBP, systolic blood pressure; DBP, diastolic blood pressure; MAP, mean arterial pressure; SpO_2_, saturation of peripheral oxygen; APS III, Acute Physiology Score III, SAPS II, Simplified Acute Physiology Score II; OASIS, Oxford Acute Severity of Illness Score; SOFA, Sequential Organ Failure Assessment; GCS, Glasgow Coma scale; SIRS, systemic inflammatory response syndrome

**Supplementary Figures**

**Fig S1** Decision curve analysis of LAR and established scoring systems for predicting 30-day mortality

LAR, lactate-to-albumin ratio; APS III, Acute Physiology Score III; SAPS II, Simplified Acute Physiology Score II; OASIS, Oxford Acute Severity of Illness Score; SOFA, Sequential Organ Failure Assessment

**Fig S2** Decision curve analysis comparing LAR (continuous) and established severity scores for predicting 30-day mortality

LAR, lactate-to-albumin ratio; APS III, Acute Physiology Score III, SAPS II, Simplified Acute Physiology Score II; OASIS, Oxford Acute Severity of Illness Score; SOFA, Sequential Organ Failure Assessment

**Fig S3** Decision curve analysis comparing LAR (categorical) and established severity scores for predicting 30-day mortality

LAR, lactate-to-albumin ratio; APS III, Acute Physiology Score III, SAPS II, Simplified Acute Physiology Score II; OASIS, Oxford Acute Severity of Illness Score; SOFA, Sequential Organ Failure Assessment
